# Increased Platelet-CD4^+^ T Cell Aggregates Are Correlated With HIV-1 Permissiveness and CD4^+^ T Cell Loss

**DOI:** 10.3389/fimmu.2021.799124

**Published:** 2021-12-20

**Authors:** Xiao-Peng Dai, Feng-Ying Wu, Cheng Cui, Xue-Jiao Liao, Yan-Mei Jiao, Chao Zhang, Jin-Wen Song, Xing Fan, Ji-Yuan Zhang, Qing He, Fu-Sheng Wang

**Affiliations:** ^1^ Medical School of Chinese People’s Liberation Army of China (PLA), Beijing, China; ^2^ Noncommissioned Officer School, Army Medical University, Shijiazhuang, China; ^3^ Department of Infectious Diseases, Peking Union Medical College Hospital, Chinese Academy of Medical Sciences & Peking Union Medical College, Beijing, China; ^4^ The Third People’s Hospital of Shenzhen, School of Medicine, Southern University of Science and Technology, Shenzhen, China; ^5^ Department of Infectious Diseases, The Fifth Medical Centre of Chinese People’s Liberation Army of China (PLA) General Hospital, National Clinical Research Center for Infectious Diseases, Beijing, China

**Keywords:** HIV-1, infection, platelet-CD4*^+^* T cell aggregates, permissiveness, T cell loss

## Abstract

Chronic HIV-1 infection is associated with persistent inflammation, which contributes to disease progression. Platelet-T cell aggregates play a critical role in maintaining inflammation. However, the phenotypic characteristics and clinical significance of platelet-CD4^+^ T cell aggregates remain unclear in different HIV-infected populations. In this study, we quantified and characterized platelet-CD4^+^ T cell aggregates in the peripheral blood of treatment-naïve HIV-1-infected individuals (TNs), immunological responders to antiretroviral therapy (IRs), immunological non-responders to antiretroviral therapy (INRs), and healthy controls (HCs). Flow cytometry analysis and immunofluorescence microscopy showed increased platelet-CD4*^+^* T cell aggregate formation in TNs compared to HCs during HIV-1 infection. However, the frequencies of platelet-CD4*^+^* T cell aggregates decreased in IRs compared to TNs, but not in INRs, which have shown severe immunological dysfunction. Platelet-CD4*^+^* T cell aggregate frequencies were positively correlated with HIV-1 viral load but negatively correlated with CD4*^+^* T cell counts and CD4/CD8 ratios. Furthermore, we observed a higher expression of CD45RO, HIV co-receptors, HIV activation/exhaustion markers in platelet-CD4*^+^* T cell aggregates, which was associated with HIV-1 permissiveness. High levels of caspase-1 and caspase-3, and low levels of Bcl-2 in platelet-CD4^+^ T cell aggregates imply the potential role in CD4^+^ T cell loss during HIV-1 infection. Furthermore, platelet-CD4*^+^* T cell aggregates contained more HIV-1 gag viral protein and HIV-1 DNA than their platelet-free CD4*^+^* T cell counterparts. The platelet-CD4*^+^* T cell aggregate levels were positively correlated with plasma sCD163 and sCD14 levels. Our findings demonstrate that platelet-CD4*^+^* T cell aggregate formation has typical characteristics of HIV-1 permissiveness and is related to immune activation during HIV-1 infection.

## Introduction

People living with human immunodeficiency virus 1 (PLWH) have higher rates of long-term comorbidities, even with successful antiretroviral therapies (ART) (1, 2). These long-term complications are related to persistent immune activation (3, 4) and the inflammatory state driven by persistent viral replication, gut microbial translocation, and co-infection (5, 6). There is a strong correlation between inflammation, coagulation, and immune activation in chronic HIV-1 infection (7). Platelets are highly specialized effector cells and are now considered the major inflammatory cells that participate in specific and nonspecific immunity, linking inflammation, chronic immune activation, and virus infection (8, 9).

It has been previously shown that platelets can directly interact with HIV-1 (10, 11). Platelets express surface receptors, such as C-type lectin-like receptor 2 (CLEC-2) and dendritic cell-specific intercellular adhesion molecule-3-grabbing non-integrin (DC-SIGN), which mediate HIV-1 binding and entry (12, 13). Platelet-derived chemokines, such as CXCL4, MIP-1α, and MIP-1β are broad-spectrum HIV-1 inhibitors that inhibit HIV-1 infection through different mechanisms (14, 15). In addition, platelets contain replication-competent HIV-1 even achieved successful ART (16–18) and serve as an acute HIV-1 reservoir during chronic HIV-1 infection (19). Previous studies have reported that platelet counts are related to HIV-1 viral load and disease progression (20). Overall, these findings indicate that platelets might modulate HIV-1 spread in patients.

HIV-1 infection can induce platelet activation, characterized by degranulation, morphological changes, and overexpression of platelet surface receptors, contributing to platelet binding to leukocytes, endothelium, and other platelets (21, 22). Platelet activation can mediate the immune response and correlates with inflammation and immune activation in HIV-infected individuals (23–27). Activated platelets release their intracellular contents and may lead to platelet-lymphocyte aggregate formation (28). Studies have shown that platelet-lymphocyte complex formation increases during HIV-1 infection (7, 29) and that the P-selectin glycoprotein ligand-1 (PSGL-1) on CD4*^+^* T cells and P-selectin (CD62P) on platelets expressed after platelet activation mediate the interactions between platelets and lymphocytes (16).

Previous studies have reported that platelet-leukocyte aggregates significantly affect cancer, infection, and other diseases (22, 30, 31). Levels of circulating platelet-leukocyte complexes correlate with markers of platelet aggregation, immune activation, and disease progression, suggesting a high risk of thromboembolism in PLWH (32, 33). At present, research on platelet-leukocyte aggregates is mainly focused on platelet-monocyte aggregates, while there are only a few studies on platelet-T cell aggregates. In HIV-1 infection, the number of platelet-T cell conjugates increases during HIV-1 infection and recruits sensitized T cells to injured sites, maintaining a state of coagulation/inflammation in PLWH (7). In addition, platelets can transmit HIV-1 to CD4*^+^* T cells *via* platelet-CD4*^+^* T cell aggregate formation, and p24 levels correlated significantly with platelet-CD4*^+^* T cell aggregate formation *in vitro* (16). Platelet binding modified the function and phenotype of lymphocytes and inhibited CD4^+^ T cells secreting IL-17 and IFN-γ in rheumatoid arthritis (34). During HIV-1 infection, activated platelets binding to CD8^+^ T cells influenced CD8^+^ T cell function by inhibiting IFN-γ production *via* TGF-β expression (35). However, the phenotypic characteristics and clinical significance of platelet-CD4*^+^* T cell aggregates are yet to be determined in HIV-1 infection.

This study compared the frequency of platelet-CD4*^+^* T cell aggregates in HIV-1-infected individuals with or without ART. Next, we focused on the phenotypic characteristics of platelet-CD4*^+^* T cell aggregates between patients infected with HIV-1 and healthy controls. Finally, we analyzed the relationship between immune activation and platelet-CD4*^+^* T cell aggregate formation during HIV-1 infection.

## Materials and Methods

### Study Participants

Our study was approved by the Ethics Committee for Clinical Research of the Fifth Medical Center of the Chinese PLA General Hospital, and blood samples were collected from participants after obtaining written informed consent in accordance with the Declaration of Helsinki. A cohort of 61 PLWH and 19 healthy controls were recruited from the Fifth Medical Center of the Chinese PLA General Hospital. Participants were divided into three groups, including 28 treatment-naïve patients (TNs), 21 immunological responders (IRs), and 12 immunological non-responders (INRs). TNs were defined as being diagnosed with chronic HIV-1 infection and mostly received ART within one week after their diagnosis. IRs were PLWH with CD4*^+^* T cell counts above 350 cells/L who had received ART for more than two years and had an undetectable viral load (<80 copies/mL) for at least two consecutive tests. INRs were PLWH with CD4*^+^* T cell counts less than 350 cells/L who had undergone ART for more than two years and had an undetectable viral load for at least two consecutive tests. The baseline characteristics of the participants are listed in **Table 1**.

**Table 1:** Baseline characteristics of the enrolled participants.

Parameter	HCs (n = 19)	TNs (n = 28)			IRs (n = 21)	INRs (n = 12)
		CD4 ≤ 200	200<CD4 ≤ 350	CD4>350		
Age (year)	33 (23-49)	39 (33-62)	29 (19-59)	43.5 (20-56)	37 (23-50)	35.5 (30-42)
Gender (M/F)	11/8	8/1	13/0	6/0	20/1	12/0
CD4^+^T cell (cells/μL)	687 (427-1013)	87 (2-195)	285 (236-334)	422.5 (373-617)	621 (373-1080)	148 (69-346)
CD8^+^T cell (cells/μL)	599 (272-1744)	995 (449-1798)	1069 (614-2618)	844 (611-2284)	718 (355-1612)	668 (382-2306)
Viral Load (log10 copies/mL)	NA	4.65 (4.12-5.36)	4.72 (3.23-5.44)	4.09 (3.13-6.10)	<LDL	<LDL
CD4/CD8 Ratio	1.24 (0.52-1.91)	0.11 (0.002-0.2)	0.28 (0.1-0.43)	0.50 (0.25-0.72)	0.73 (0.33-1.79)	0.23 (0.09-0.51)

Data are expressed as median (range). HC, healthy controls; TNs, treatment-naïve HIV-1-infected individuals; IRs, immunologic responders receiving successful ART; INRs, immune non-responders to ART; n, number of individuals per group; NA, not applicable; M, male; F, female; LDL, lower detection limit. TNs are divided into three subgroups according to blood CD4^+^ T cell count.

### Isolation of PBMCs and Flow Cytometric Analysis of Platelet-CD4*^+^* T Cell Aggregates

Peripheral blood mononuclear cells (PBMCs) were isolated from the peripheral blood in EDTA-K_2_ tubes *via* density gradient centrifugation. PBMCs were labeled with fluorescently conjugated antibodies or reagents for the specific detection of markers on the surface or in the cytoplasm of cells, according to the manufacturer’s instructions. The fluorescently conjugated antibodies or reagents used include: anti-human CD3 (OKT3), anti-human CD4 (OKT4), anti-human CD45RO (UCHL1), anti-human CXCR4 (12G5), anti-human CCR5 (J418F1), and anti-human CD62P (AK4), anti-Bcl-2 (100), which were bought from Biolegend, San Diego, CA, USA; anti-human CD42a (ALMA.16), anti-human CD38 (HIT2), anti-human HLA-DR (L243), and anti-active Caspase-3 (C92-605) purchased from BD Biosciences, Franklin Lakes, New Jersey, USA; anti-human PD-1 (MIH4) provided by eBioscience, San Diego, CA, USA; anti-P24 (KC57) from Beckman Coulter, Kraemer Boulevard Brea, CA, USA; and a FAM-FLICA^®^ Caspase-1 Assay Kit from ImmunoChemistry, Bloomington, MN, USA. Data were acquired using a BD Canto II flow cytometer (BD Biosciences) and analyzed using FlowJo software (Tree Star, Woodburn, Oregon, USA).

### Cell Sorting

PBMCs were stained using anti-human CD3, anti-human CD4, and anti-human CD42a at 4°C for 30 min, and then sorted into CD42a*^+^* CD4*^+^* T cells and CD42a^-^CD4*^+^* T cells using a MoFLo High-Performance Cell Sorter (Cytomation, CA, USA). The sorted cell population purity exceeded 95% in all sorted samples.

### HIV-1 DNA Quantification

Total cellular DNA was extracted from sorted CD42a*^+^* CD4*^+^* T cells and CD42a^-^CD4*^+^* T cells using Qiagen QIAsymphony DNA Mini Kits (Qiagen, Hilden, Germany). The level of HIV-1 DNA was determined using a fluorescence-based real-time SUPBIO HIV-1 Quantitative Detection Kit (SUPBIO, Guangzhou, China) using a QuantStudio Dx Real-Time PCR Instrument (Biosystems, Barcelona, Spain). The actin gene was simultaneously detected using the same PCR conditions to quantify the cell number.

### Imaging Flow Cytometry

Platelet-CD4*^+^* T cell aggregate formation and HIV-1 P24 expression were detected using an ImageStreamX Mark II Imaging Flow Cytometer (Merck Millipore, Billerica, MA, USA). To detect platelet-CD4*^+^* T cell aggregates, 1×10^6^ PBMCs were resuspended in 100 μL PBS containing 2% BSA (Sigma, St. Louis, Missouri, USA) and stained at 4°C for 30 min. To visualize HIV-1 P24 in platelet-CD4*^+^* T cell aggregates, PBMCs were first stained with anti-CD3, anti-CD4, and anti-CD42 antibodies, then permeabilized using a Cytofix/Cytoperm Kit (BD Biosciences) and finally stained with anti-P24 antibodies at 4°C for 45 min. Stained cells were resuspended in 50 μL PBS and analyzed using an ImageStreamX Mark II Imaging Flow Cytometer. All graphs were generated using the IDEAS software.

### Immunohistochemistry

Paraffin-embedded sections of formalin-fixed lymph node biopsies (5 μm) were blocked using 0.3% H_2_O_2_ and placed on APES-coated slides. Antigens were cooked for 3 min in citrate buffer (Solarbio Life Sciences, Beijing, China) to achieve retrieval. Mouse monoclonal anti-human CD42a antibody (ThermoFisher, Waltham, Massachusetts, USA) and rabbit monoclonal anti-human CD4 antibody (Zhongshan Goldenbridge Biotech, Beijing, China) were added to the slides at 4°C overnight. 4’,6-diamidino-2-phenylindole (DAPI, blue color) was used to stain the nuclei as previously described (36). Images were obtained using a Leica Biosystems Aperio VERSA scanning system (Leica, Richmond, Virginia, USA).

### ELISA for the Detection of Plasma Cytokines

The plasma concentrations of soluble CD14 (sCD14) and soluble CD163 (sCD163) were detected using standard ELISA kits according to the manufacturer’s instructions (R&D Systems, Minneapolis, MN, USA).

### Statistical Analysis

Statistical analyses were performed using the GraphPad Prism software version 7. Comparisons were performed for two paired groups using Wilcoxon’s paired test and two unpaired groups using the Mann-Whitney U test. For three or more groups, Dunn’s multiple comparisons test was performed for comparison with every other group when the Kruskal-Wallis test was used to determine significant differences among groups. Correlation coefficients were calculated for parametric distributions using Pearson’s correlation test and nonparametric distributions using Spearman’s correlation test. The results were considered significant at *p*<0.05. *P < 0.05, **P ≤ 0.01, ***P ≤ 0.001, ****P ≤ 0.0001.

## Results

### An Increase in Platelet-CD4*^+^* T Cell Aggregates Is Related to Disease Progression and Clinical Outcomes During Chronic HIV-1 Infection

**Figure 1:**
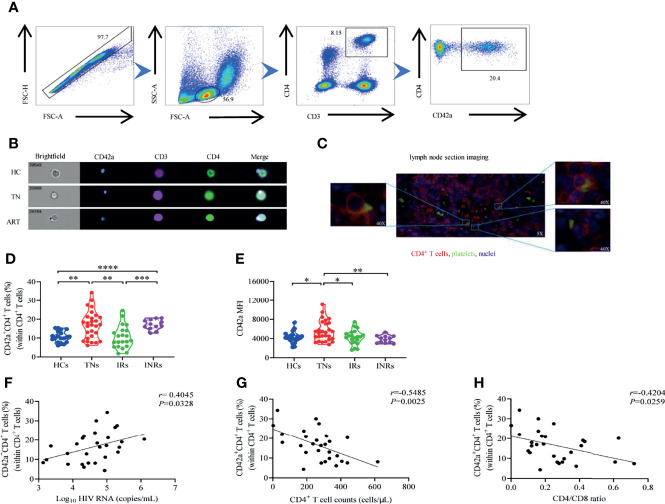
Detection of platelet-CD4*^+^* T cell aggregates in HCs, TNs, IRs, and INRs. **(A)** Representative gating strategy for the flow cytometry analysis of platelet-CD4*^+^* T cell aggregates and platelet-free CD4*^+^* T cells from a TN patient. CD42a*^+^* CD4*^+^* T cells represent platelet-CD4*^+^* T cell aggregates while CD42a^-^CD4*^+^* T cells represent platelet-free CD4*^+^* T cells. **(B)** Representative image of circulating platelet-CD4*^+^* T cell aggregates that came separately from HC and HIV-1-infected patients with or without ART as obtained from imaging flow cytometry. Platelets are recognized by anti-CD42a FITC (blue) while CD4*^+^* T cells are recognized by anti-human CD3 BV510 (purple) and anti-human CD4 BV421 (green). **(C)** Representative images of platelet-CD4*^+^* T cell aggregates in lymph nodes from an HIV-1-infected patient without ART as visualized *via* immunofluorescence microscopy. **(D)** Comparison of platelet-CD4*^+^* T cell aggregate frequencies in CD4*^+^* T cells from HCs (n=19), TNs (n=28), IRs (n=21) and INRs (n=12). **(E)** Median fluorescence intensity (MFI) values of CD42a were measured in all four groups. **(F–H)** Correlation between **(F)** HIV-1 viral load, **(G)** absolute CD4*^+^* T cell counts, and **(H)** CD4/CD8 ratio with platelet-CD4*^+^* T cell aggregate frequencies in TNs (n=28). *P < 0.05, **P ≤ 0.01, ***P ≤ 0.001, ****P ≤ 0.0001.

CD42a is a specific marker expressed on platelets. Platelet-monocyte aggregates are determined by co-expressing CD42a and CD14 (37). In our study, the platelet-CD4*^+^* T cell aggregates in CD4*^+^* T cells stained with antibodies against CD4^+^ T cells (anti-CD4/anti-CD3) and antibodies against platelets (anti-CD42a) were examined *via* flow cytometry. A representative gating strategy is shown in **Figure 1A**. Circulating CD4*^+^* T cells were initially divided into two populations, CD42a*^+^* and CD42a^-^, which were identified as platelet-CD4*^+^* T cell aggregates and platelet-free CD4*^+^* T cells, respectively. To directly visualize platelet-CD4*^+^* T cell aggregate formation, we utilized flow cytometry and immunofluorescence imaging to confirm platelet-CD4*^+^* T cell aggregates in peripheral blood and lymph nodes in PLWH, respectively. We observed platelet-CD4*^+^* T cell aggregate formation in PBMCs isolated from HCs and HIV-1-infected patients with or without ART (**Figure 1B**). In addition, platelet-CD4*^+^* T cell aggregate formation was also observed in lymph nodes from a TN patient (**Figure 1C**). Overall, we observed platelet-CD4*^+^* T cell aggregate formation in PLWH.

We first compared the proportions of circulating platelet-CD4*^+^* T cell aggregates from HCs (n=19), TNs (n=28), IRs (n=21), and INRs (n=12). The demographic and clinical characteristics of the patients are shown in **Table 1**. The proportions of platelet-CD4*^+^* T cell aggregates were significantly increased in both TNs and INRs compared to those in HCs (*P*<0.01 and *P*<0.0001, respectively). However, the proportions of platelet-CD4*^+^* T cell aggregates in IRs were lower than those in TNs (*P*<0.01) and INRs (*P*<0.001) but comparable to those in HCs (*P*>0.05) (**Figure 1D**). In addition, platelet-CD4*^+^* T cell aggregates within the samples obtained from TNs expressed more CD42a as measured from the median fluorescence intensity (MFI) values of CD42a compared to those from the HCs and HIV-1-infected patients with ART, including IRs and INRs (*P*<0.05 for HCs, *P*<0.05 for IRs, and *P*<0.01 for INRs, respectively) (**Figure 1E**). These results indicate an increased number of platelets bound to CD4*^+^* T cells during HIV-1 infection but were compromised after ART commencement.

We further analyzed the relationship between platelet-CD4*^+^* T cell aggregate frequencies and disease progression in TNs. The viral load (log10 HIV-1 RNA copies/mL), CD4*^+^* T cell counts and CD4/CD8 ratio were quantified and calculated in peripheral blood obtained from TNs. The frequencies of platelet-CD4*^+^* T cell aggregates in CD4*^+^* T cells were positively correlated with plasma HIV-1 RNA (*r*=0.4045, *P*=0.0328), but were negatively correlated with CD4*^+^* T cell counts and CD4/CD8 ratio in TNs (*r*=0.5485, *P*=0.0025 and *r*=0.4204, *P*=0.0259, respectively) (**Figures 1F–H**).

These data suggest that the increased frequencies of platelet-CD4*^+^* T cell aggregates could be associated with HIV-1 disease progression and clinical outcomes in HIV-1-infected patients.

### Phenotypic Characteristics of Platelet-CD4*^+^* T Cell Aggregates

**Figure 2:**
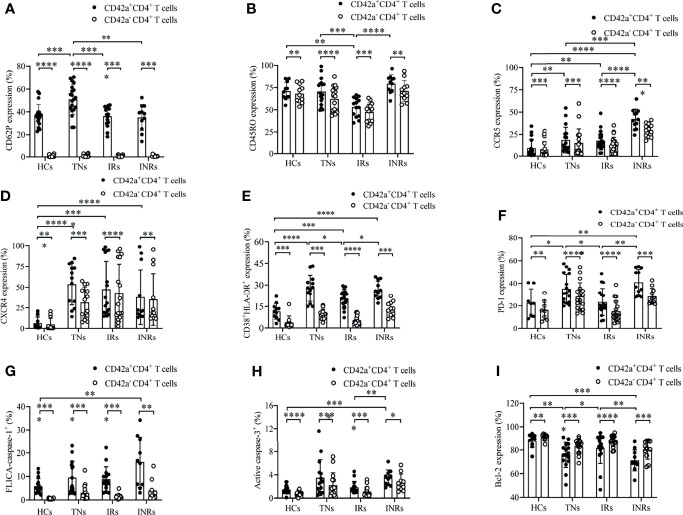
Phenotype of platelet-CD4*^+^* T cell aggregates in HCs, TNs, IRs, and INRs. The phenotypic characteristics of platelet-CD4*^+^* T cell aggregates were analyzed, including platelet activation, CD45RO expression, HIV-1 co-receptors, immune checkpoint receptors, activation markers, and pyroptosis/apoptosis markers, as well as anti-apoptotic proteins from HCs, TNs, IRs, and INRs. Platelet-CD4^+^ T cell aggregates and platelet-free CD4^+^ T cells were compared with regards to their expression of CD62P **(A)** [HCs (n=19), TNs (n=20), IRs (n=16), and INRs (n=11)], CD45RO **(B)** [HCs (n=11), TNs (n=18), IRs (n=14), and INRs (n=11)], CCR5 **(C)** [HCs (n=17), TNs (n=18), IRs (n=21), and INRs (n=12)], CXCR4 **(D)** [HCs (n=13), TNs (n=13), IRs (n=17), and INRs (n=12)], CD38/HLA-DR **(E)** [HCs (n=11), TNs (n=13), IRs (n=17), and INRs (n=11)], PD-1 **(F)** [HCs (n=8), TNs (n=17), IRs (n=17), and INRs (n=11)], caspase-1 **(G)** [HCs (n=16), TNs (n=15), IRs (n=15), and INRs (n=10)], caspase-3 **(H)** [HCs (n=16), TNs (n=15), IRs (n=16), and INRs (n=10)], and Bcl-2 **(I)** [HCs (n=10), TNs (n=16), IRs (n=16) and INRs (n=11)]. *P < 0.05, **P ≤ 0.01, ***P ≤ 0.001, ****P ≤ 0.0001.

Platelet binding influences the phenotype of T lymphocytes in rheumatoid arthritis (38). However, the phenotypic characteristics of platelet-CD4*^+^* T cell aggregates during HIV-1 infection has not been fully defined. To address this problem, we studied the phenotypic characteristics of platelets in platelet-CD4*^+^* T cell aggregates in PLWH. We first investigated the activation of platelets in platelet-CD4*^+^* T cell aggregates by analyzing CD62P expression, a marker of platelet adhesion and activation (39), from HCs, TNs, IRs, and INRs. CD62P expression levels were increased in platelet-CD4*^+^* T cell aggregates in TNs (*P*<0.0001) and decreased in IRs and INRs who had received successful ART (*P*<0.0001 and *P*<0.001, respectively) (**Figure 2A** and **Supplementary Figure 1A**).

We further analyzed the phenotypic characteristics of CD4*^+^* T cells in platelet-CD4*^+^* T cell aggregates. Studies have demonstrated that activated CD4^+^ T cells identified by CD45RO expression are the predominantly infected cells during HIV-1 infection (40–42). Analysis of CD45RO expression levels on platelet-CD4*^+^* T cell aggregates using flow cytometry revealed that platelet-CD4*^+^* T cell aggregates expressed more CD45RO than their counterpart platelet-free CD4*^+^* T cells (*P*<0.01 for HCs, *P*<0.0001 for TNs, *P*<0.001 for IRs, and *P*<0.01 for INRs), indicating that HIV-1 may preferentially targets and persists in platelet-CD4^+^ T cell aggregates. (**Figure 2B** and **Supplementary Figure 1B**).

Platelets transmit HIV-1 to CD4*^+^* T cells *via* platelet-CD4*^+^* T cell aggregate formation (16). To further explore the permissiveness of platelet-CD4*^+^* T cell aggregates to HIV-1, we detected HIV-1 co-receptor expression, which mediates HIV-1 entry processes, *via* flow cytometry. Both CCR5 and CXCR4 levels were higher in platelet-CD4*^+^* T cell aggregates than their counterpart platelet-free CD4*^+^* T cells in all groups involved (*P*<0.001 for TNs, *P*<0.0001 for IRs, and *P*<0.001 for INRs; *P*<0.001 for TNs, *P*<0.0001 for IRs and *P*<0.01 for INRs, respectively). Furthermore, both CCR5 and CXCR4 expression levels were elevated in PLWH compared to HCs (*P*<0.01 for TNs, *P*<0.01 for IRs, and *P*<0.0001 for INRs; *P*<0.0001 for TNs, *P*<0.0001 for IRs, and *P*<0.0001 for INRs, respectively). Notably, the INRs had the highest expression of CCR5 compared to other groups (*P*<0.0001 for HCs, *P*<0.001 for TNs, and *P*<0.0001 for IRs) (**Figures 2C, D** and **Supplementary Figures 1C, D**).

Since productive HIV-1 infection depends on T cell activation (43), we investigated the expression of markers of T cell activation in the different groups. As shown in **Figure 2E**, platelet-CD4*^+^* T cell aggregates showed higher levels of activation than their counterpart platelet-free CD4*^+^* T cells in all groups, as detected from CD38 and HLA-DR co-expression analysis (*P*<0.001 for HCs, *P*<0.001 for TNs, *P*<0.0001 for IRs, and *P*<0.001 for INRs). Notably, highly activated platelet-CD4*^+^* T cell aggregates were enriched in HIV-1-infected patients whether treated with ART or not. Compared to TNs, it was partially restored after ART in IRs, not INRs, but did not return to normal levels in HCs (**Figure 2E** and **Supplementary Figure 1E**).

Immune checkpoints, such as PD-1, are preferentially expressed on CD4*^+^* T cells containing integrated HIV-1 DNA, induced by T cell activation (44–46). Next, we measured the expression of PD-1 in platelet-CD4*^+^* T cell aggregates and platelet-free CD4*^+^* T cells. Platelet-CD4*^+^* T cell aggregates expressed slightly higher levels of PD-1 than their counterparts in all groups (HCs, *P*<0.01; *P*<0.0001; IRs, *P*<0.0001; INRs, *P*<0.001). Compared to TNs, platelet-CD4*^+^* T cell aggregate proportions expressing PD-1 were partially restored in IRs, not INRs, but did not return to normal levels in HCs (**Figure 2F** and **Supplementary Figure 1F**).

Virus infection and replication are related to programmed cell death (47). Caspase-1 dependent pyroptosis and caspase-3 dependent apoptosis exert an important effect on CD4*^+^* T cell loss in PLWH (48). The percentage of caspase-1 and caspase-3 expression was remarkably higher on platelet-CD4*^+^* T cell aggregates than in their counterparts in all groups (*P*<0.0001 for HCs, *P*<0.0001 for TNs, *P*<0.0001 for IRs, and *P*<0.01 for INRs; *P*<0.0001 for HCs, *P*<0.001 for TNs, *P*<0.0001 for IRs, and *P*<0.05 for INRs, respectively) (**Figures 2G, H** and **Supplementary Figures 1G, H**).

The anti-apoptosis molecule Bcl-2 is an important regulator of cellular survival (49). Reports have shown that high levels of HIV-1 replication correlate with the downregulation of circulating Bcl-2 protein in PLWH (50). Next, we detected Bcl-2 expression in platelet-CD4*^+^* T cell aggregates and their counterparts. The results showed that the percentage of Bcl-2 expression was remarkably lower in platelet-CD4*^+^* T cell aggregates than in their counterparts in all groups (*P*<0.01 for HCs, *P*<0.0001 for TNs, *P*<0.0001 for IRs, and *P*<0.001 for INRs). In addition, the percentages of platelet-CD4*^+^* T cell aggregates expressing Bcl-2 in TNs were decreased compared to HCs (*P*<0.01), were increased in IRs compared to HCs (*P*<0.05), but not in INRs with ART (**Figure 2I** and **Supplementary Figure 1I**).

In general, our study demonstrated that platelet-CD4*^+^* T cell aggregates have a high level of CD45RO and are primarily of activation and cell death characteristics; thus, these characteristics increase the permissiveness of platelet-CD4^+^ T cell aggregates to HIV-1 infection and may be involved in CD4^+^ T cell loss.

### Elevated Levels of HIV p24 and DNA in Platelet-CD4*^+^* T Cell Aggregates

**Figure 3:**
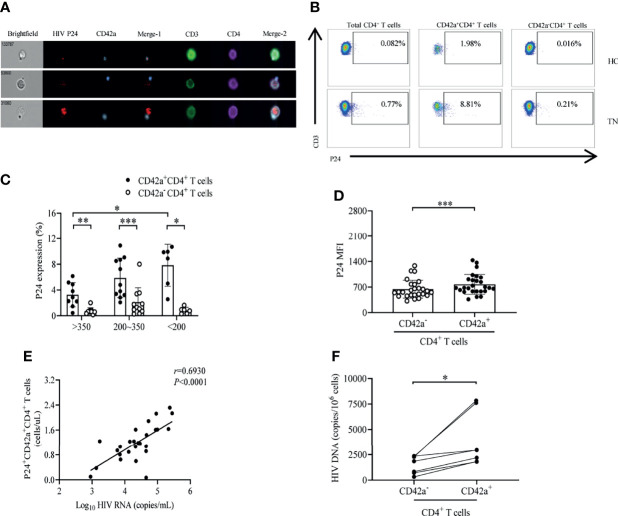
Elevated levels of HIV-1 p24 and DNA in platelet-CD4^+^ T cell aggregates. **(A)** Representative images of p24 expression in platelet-CD4^+^ T cell aggregates *via* imaging flow cytometry from a treatment-naïve patient. Top row: p24 visualized only in the platelets of platelet-CD4^+^ T cell aggregates. Middle row: p24 visualized only in the CD4^+^ T cells. Bottom row: p24 visualized in both platelets and CD4^+^ T cells. **(B)** Representative gating strategy for p24 expression in platelet-CD4^+^ T cell aggregates and their counterparts from TNs and HCs. **(C)** P24 expression in platelet-CD4^+^ T cell aggregates and their counterparts in different groups of treatment-naïve HIV-1-infected subjects. CD4^+^ T cell counts>350 cells/μL (n=9), 200≤CD4^+^ T cell counts ≤ 350 cells/μL (n=11), CD4^+^ T cell counts<200 cells/μL (n=6). **(D)** Median fluorescence intensity (MFI) values of p24 were compared between platelet-CD4^+^ T cell aggregates and their counterparts in TNs (n=26). **(E)** Correlation between HIV-1 viral load and platelet-CD4^+^ T cell aggregates expressing p24 in TNs (n=26). **(F)** Platelet-CD4^+^ T cell aggregates and their counterparts were isolated from PBMCs of IRs (n=7); HIV-1 DNA was detected in these two cell fractions. *P < 0.05, **P ≤ 0.01, ***P ≤ 0.001.

We have shown that platelet-CD4*^+^* T cell aggregates displayed a typical phenotype of HIV-1 permissiveness. It has been found that platelets harbor infectious HIV-1 despite viral suppression and can transmit HIV-1 to CD4*^+^* T cells to induce CD4*^+^* T cell infection by activating platelets and forming platelet-CD4*^+^* T cell aggregates (16, 19). Next, we analyzed the expression of viral proteins and HIV-1 DNA in platelet-CD4*^+^* T cell aggregates. We isolated PBMCs from treatment-naïve patients and performed imaging flow cytometry with immunostaining for CD42a to detect platelets, CD3/CD4 to detect CD4*^+^* T cells, and p24 to detect HIV-1 gag viral protein visually. Platelet-CD4*^+^* T cell aggregates were found to contain HIV-1 viral protein; HIV-1 gag protein could not only exist in platelets or in the CD4*^+^* T cells of platelet-CD4*^+^* T cell aggregates alone, but also simultaneously reside in both platelets and CD4*^+^* T cells of platelet-CD4*^+^* T cell aggregates (**Figure 3A**). Next, HIV-1 infection in platelet-CD4*^+^* T cell aggregates was analyzed based on intracellular HIV-1 p24 expression. A representative gating strategy used to identify HIV-1 p24 production in different CD4*^+^* T cell populations is shown in **Figure 3B**. The expression level of HIV-1 p24 was increased significantly in platelet-CD4*^+^* T cell aggregates compared to their counterparts in each group. Most notably, platelet-CD4*^+^* T cell aggregates in the group with CD4*^+^* T cell counts below 200 cells/μL contained more HIV-1 p24 than those in the group with CD4*^+^* T cell counts above 350 cells/μL (*P*<0.05) (**Figure 3C**). Furthermore, there was an increase in intracellular HIV-1 p24 on platelet-CD4*^+^* T cell aggregates in comparison with their counterparts as measured using the MFI (*P*<0.001) (**Figure 3D**). The number of HIV-1 p24-producing platelet-CD4*^+^* T cell aggregates from the PBMCs of treatment-naïve patients was significantly positively correlated with the individual viral load (**Figure 3E**) (*r*=0.6930, *P*<0.0001). Additionally, platelet-CD4*^+^* T cell aggregates showed more HIV-1 DNA than their counterparts (**Figure 3F**) in IRs (*P*<0.05).

### Increased Platelet-CD4*^+^* T Cell Aggregates Correlate With sCD14 and sCD163

**Figure 4:**
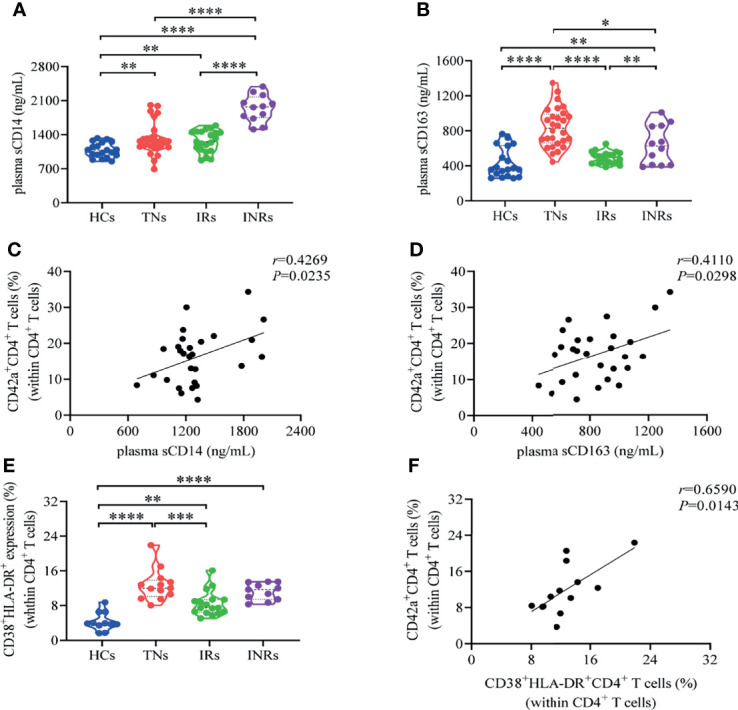
An increased frequency of platelet-CD4*^+^* T cell aggregates is positively correlated with sCD14 and sCD163 during HIV-1 infection. **(A)** Serum level of sCD14 from HCs (n=19), TNs (n=28), IRs (n=21) and INRs (n=12). **(B)** Serum level of sCD163 from HCs (n=19), TNs (n=28), IRs (n=21) and INRs (n=12). **(E)** Frequencies of CD38^+^HLA-DR^+^CD4^+^T cell in CD4^+^ T cells from HCs (n=11), TNs (n=13), IRs (n=17) and INRs (n=11). Correlation between **(C)** plasma sCD14 levels (n=28), **(D)** plasma sCD163 levels (n=28), and **(F)** CD38^+^HLA-DR^+^CD4^+^ T cells (n=13) and the frequencies of platelet-CD4^+^ T cell aggregates in CD4^+^ T cells. *P < 0.05, **P ≤ 0.01, ***P ≤ 0.001, ****P ≤ 0.0001.

We have demonstrated that HIV-1 infection promotes the formation of platelet-CD4*^+^* T cell aggregates, and the upregulation of CD62P could partly explain the elevated frequencies of platelet-CD4*^+^* T cell aggregates in patients infected with HIV-1. Studies have reported that increased immune activation may drive the formation of platelet-monocyte during HIV-1 infection (33, 51–53). To further determine the relationship of immune activation and platelet-CD4*^+^* T cell aggregate formation, we quantified the expression of soluble CD14 (sCD14) and soluble CD163 (sCD163) in plasma. Results showed that plasma sCD14 levels were elevated in HIV-1-infected patients with or without ART compared to HCs (*P*<0.01 for TNs, *P*<0.01 for IRs, and *P*<0.0001 for INRs) (**Figure 4A**). Similarly, plasma sCD163 levels were also elevated in TNs and INRs compared to HCs (*P*<0.0001 for TNs and *P*<0.01 for INRs, respectively), and ART restored sCD163 levels in IRs (**Figure 4B**). In addition, the expression of sCD14 and sCD163 was positively correlated with the frequency of platelet-CD4*^+^* T cell aggregates in TNs (*r*=0.4269, *P*=0.0235, and *r*=0.4110, *P*=0.0298, respectively) (**Figures 4C, D**).

Reports have shown that T cell activation significantly promotes the formation of platelet-T cells (54). We further analyzed CD4*^+^* T cell activation frequency defined by CD38 and HLA-DR co-expression in all four groups. The results showed that the frequencies of activated CD4*^+^* T cells were markedly increased in HIV-1-infected patients compared to HCs (*P*<0.0001 for TNs, *P*<0.001 for IRs, and *P*<0.0001 for INRs), and could be partly restored by ART in IRs compared to TNs (*P*<0.05), but did not return to normal levels in HCs (**Figure 4E**). Notably, platelet-CD4*^+^* T cell aggregate frequencies were positively correlated with activated CD4*^+^* T cells in TNs (*r*=0.5604, *P*=0.0499) (**Figure 4F**). Altogether, these findings indicate that increased platelet-CD4*^+^* T cell aggregate formation may have potential connection with immune activation in PLWH.

## Discussion

Platelets are the chief effector cells that link hemostatic and immune responses. The interaction between coagulation and immunity is essential for the antiviral immune response. Platelet-leukocyte aggregates have been previously reported in cancer, psoriasis, rheumatoid arthritis, and multiple sclerosis (22, 31, 38, 55). It has been demonstrated that platelet-monocyte aggregate formation imparts a higher risk of thromboembolism in PLWH (33). Activated platelet-T cell aggregates affect the recruitment of antigen-experienced T cells to injured organs and tissues, contributing greatly to coagulation and inflammation during HIV-1 infection (7). In this study, we directly visualized platelet-CD4*^+^* T cell aggregate formation in both peripheral blood and lymph nodes in different HIV-1 infected populations. To the best of our knowledge, this is the first time that platelet-CD4^+^ T cell aggregates were observed directly *via* imaging flow cytometry in the peripheral blood and lymph nodes of PLWH. Platelet-CD4*^+^* T cell aggregate formation was increased during HIV-1 infection. These findings are consistent with the interesting work reported by Green et al., which demonstrated that there was an increased frequency of platelet-CD4*^+^* T cell aggregates in PLWH with suppressed viremia (7). Patients with poor CD4*^+^* T cell recovery demonstrated increased numbers of HIV-1*^+^* platelets in PLWH (19). In this study, we demonstrated that HIV-1 gag protein not only exists in the platelets or CD4*^+^* T cells in platelet-CD4*^+^* T cell aggregates on their own, but also simultaneously resides in both platelets and CD4*^+^* T cells in platelet-CD4*^+^* T cell aggregates. Platelets serve as an acute HIV-1 reservoir and a carrier to fuel the HIV-1 infection of CD4*^+^* T cells through the formation of platelet-CD4*^+^* T cell aggregates under co-culture conditions (16, 19). We demonstrated that platelet-CD4*^+^* T cell aggregates, compared with platelet-free CD4*^+^* T cells, harbored higher expression of p24 in TNs and higher levels of integrated HIV-1 DNA in IRs. Furthermore, platelet-CD4*^+^* T cell aggregate frequency decreased after ART in IRs, but not in INRs. Our observations also demonstrated that these aggregates were positively correlated with the viral load but were negatively correlated with CD4*^+^* T cell counts and the CD4/CD8 ratio. Hence, platelet-CD4^+^ T cell aggregates may be related to HIV-1 disease progression and clinical outcomes in HIV-1-infected patients.

Next, we analyzed the phenotype of platelet-CD4*^+^* T cell aggregates in patients infected with HIV-1. Previous reports have demonstrated that HIV-1-infected patients display elevated platelet activation with or without ART (28, 56). The activation state of platelets in platelet-CD4*^+^* T cell aggregates was analyzed by detecting CD62P expression. Strikingly, CD62P expression levels were increased on platelet-CD4^+^ T cell aggregates in TNs but decreased after ART. CD62P plays an important role in recruiting lymphocytes to inflamed peripheral tissues. The presence of platelet-CD4*^+^* T cell aggregates in lymph nodes may be mediated by activated platelets, contributing to the antiviral role of T cells in the lymph nodes during HIV-1 infection. It has been reported that activated platelets are more likely to form platelet-CD4*^+^* T cell aggregates, and that platelets selectively bind to larger and activated lymphocytes (54, 55). Meagan et al. demonstrated that heightened platelet activation is attenuated by 1 week of aspirin therapy in treated HIV-1 patients (57). Thus, it is reasonable to speculate that aspirin and/or other antithrombotic drugs may reduce the formation of platelet-CD4^+^ T cell aggregates through reducing platelet activation in HIV-1 patients. Further research is needed to confirm this speculation. In our study, the activation of CD4^+^ T cells was positively related to the increased formation of platelet-CD4^+^ T cell aggregates. Thus, the increased activation of platelets and CD4^+^ T cells could partially explain the elevated formation of platelet-CD4*^+^* T cell aggregates during HIV-1 infection. PSGL-1 is constitutively expressed at high levels on all T cells, however the binding affinity for its ligand is determined by the degree of glycosylation of PSGL-1, which is mediated by glycosytransferases and tyrosine sulphotransferases (58, 59). In addition to CD62P-PSGL-1, other ligand-receptor ligations are involved in platelet-lymphocyte formation: GPIb-CD11b, CD40-CD40L, GPIIb/IIIa-CD11/CD18 (60). Further research is needed to confirm the molecular mechanisms of platelet-CD4^+^ T cell aggregate formation.

Our results demonstrate a direct interaction between platelets and CD4^+^ T cells during HIV-1 infection. Direct binding to CD4^+^ T cells by platelets may also affect the phenotype and function of CD4*^+^* T cells in platelet-CD4*^+^* T cell aggregates. Studies have shown that activated CD4^+^ T cells identified by CD45RO expression have a high affinity for CD62P and are preferentially targeted by HIV-1 (7, 61). Our results showed that platelet-CD4*^+^* T cell aggregates have a higher expression of CD45RO than their counterparts, which indicates their potential capability of binding to platelets and infection by HIV-1. The HIV-1 co-receptors CXCR4 and CCR5 mediate the infection of CD4*^+^* T cells by HIV-1 (62). The chemokine receptors CCR5 and CXCR4 are the principal co-receptors for the R5-dependent and X4-dependent strains of HIV, respectively. An elevated expression of the HIV-1 co-receptors promotes HIV-1 permissiveness. In this study, CCR5 and CXCR4 were both overexpressed on platelet-CD4*^+^* T cell aggregates compared to their counterparts and were upregulated during HIV-1 infection, indicating that these cells were more permissive to R5 and X4 HIV-1 strains. Productively infected cells have a higher expression of activation markers and immune checkpoint molecules (43, 45, 63, 64). Our results demonstrated that platelet-CD4*^+^* T cell aggregates expressed higher CD38/HLA-DR and PD-1 levels than their counterparts in all groups, suggesting the potential permissiveness of platelet-CD4*^+^* T cell aggregates to HIV-1 infection.

It is generally known that virus infection and replication are related to programmed cell death, which is the cause of many infection-induced pathological changes (47). PBMCs with high levels of viral replication are more sensitive to apoptosis (50). Bcl-2 is an apoptosis-related gene that plays an important anti-apoptotic role. High levels of HIV-1 replication are correlated with the downregulation of Bcl-2 protein in PLWH (50). We demonstrated that platelet-CD4*^+^* T cell aggregates had higher levels of caspase-1 and caspase-3 and a lower level of Bcl-2. These phenotypic characteristics of programmed cell death also indicated that platelet-CD4*^+^* T cell aggregates displayed typical characteristics of HIV-1 permissiveness. HIV-1 infection leads to a progressive loss of CD4^+^ T cells, which may be due to the viral cytopathic effect in productively infected CD4^+^ T cells and the bystander effect in uninfected CD4^+^ T cells (48). During HIV-1 infection, programmed cell death, including caspase-3-dependent apoptosis and caspase-1-dependent pyroptosis, contributes to CD4^+^ T cell loss and immunopathogenesis (65). In our study, increased platelet-CD4^+^ T cell aggregates were negatively correlated with CD4^+^ T cell counts. Furthermore, high levels of caspase-1 and caspase-3, and low levels of anti-apoptotic protein Bcl-2 in platelet-CD4^+^ T cell aggregates imply that these aggregates are more prone to apoptosis and pyroptosis. Hence, elevated expression of caspase-1 and caspase-3 and downregulated expression of Bcl-2 in platelet-CD4^+^ T cell aggregates might serve as a potential mechanism for driving CD4^+^ T cell loss.

Reports have shown that the formation of platelet-monocyte aggregates is positively correlated with sCD163 levels during HIV-1 infection (33). In this study, the expression of sCD14 and sCD163 was upregulated and positively correlated with elevated frequency of platelet-CD4^+^ T cell aggregates in TNs. Furthermore, we found frequencies of activated CD4^+^ T cells (CD38^+^ HLA-DR^+^) were markedly increased in TNs compared to HCs, and could be partly restored by ART in IRs compared to TNs, but did not return to normal levels in HCs. This phenomenon is much like levels of chronic immune activation in HIV-1 patients. Furthermore, platelet-CD4^+^ T cell aggregate frequencies were positively correlated with activated CD4^+^ T cells in TNs. However, we do not know for sure whether platelet-CD4^+^ T aggregate formation induces immune activation or whether immune activation promotes platelet-CD4^+^ T aggregate formation. Further studies examining these causal relationships are warranted. HIV-1 infected individuals have higher rates of long-term comorbidities, such as cardiovascular disease (CVD), which are related to persistent immune activation and inflammatory state driven by persistent virus replication. Elevated sCD14 and sCD163 levels are associated with a higher risk of CVD and serve as markers of HIV disease progression (66–68). While our results have implications for the role of platelet-CD4^+^ T cell aggregates in HIV-1 infection, further studies are needed to establish their potential function in HIV-1 related comorbidities during HIV-1 infection.

In summary, we identified platelet-CD4*^+^* T cell aggregates both in the peripheral blood and lymph nodes of PLWH and revealed significant alterations during HIV-1 infection. The activation of platelets and CD4*^+^* T cells may promote the formation of platelet-CD4*^+^* T cell aggregates. CD4*^+^* T cell interactions with activated platelets exhibited phenotypic characteristics with permissive to HIV-1 infection and was related to immune activation during HIV-1 infection. High levels of caspase-1 and caspase-3, and low levels of Bcl-2 in platelet-CD4^+^ T cell aggregates imply the potential role in CD4^+^ T cell loss during HIV-1 infection. However, our study had some limitations. First, the sample size, especially the INRs included, was relatively small. Further, we were unsure whether the higher expression of p24 or HIV-1 DNA in platelet-CD4*^+^* T cell aggregates was derived from platelets or CD4*^+^* T cells. Further studies designed to separate platelet-CD4*^+^* T cell aggregates *ex vivo* may provide insights into the mechanisms of platelet-CD4*^+^* T cell aggregate permissiveness and disease progression in HIV-1 infection.

## Data Availability Statement

The original contributions presented in the study are included in the article/**Supplementary Material**. Further inquiries can be directed to the corresponding authors.

## Ethics Statement

The studies involving human participants were reviewed and approved by the Ethics Committee for Clinical Research of The Fifth Medical Center of PLA General Hospital. The patients/participants provided their written informed consent to participate in this study.

## Author Contributions

J-YZ, QH, and F-SW designed research. X-PD, F-YW, and CC performed research. X-PD, F-YW, CC, X-JL, Y-MJ, CZ, J-WS, and XF wrote the paper. X-PD, F-YW, and CC analyzed data. J-YZ, QH, and F-SW supervised the project. All authors contributed to the article and approved the submitted version.

## Funding

This research was supported by Peking University Clinical Scientist Program Special (BMU2019LCKXJ013), National Natural Science Foundation Innovation Research Group Project (81721002), and the Sanming Project of Medicine Project in Shenzhen (grant number SZSM201612014).

## Conflict of Interest

The authors declare that the research was conducted in the absence of any commercial or financial relationships that could be construed as a potential conflict of interest.

## Publisher’s Note

All claims expressed in this article are solely those of the authors and do not necessarily represent those of their affiliated organizations, or those of the publisher, the editors and the reviewers. Any product that may be evaluated in this article, or claim that may be made by its manufacturer, is not guaranteed or endorsed by the publisher.
