# Supplementary material for: Increased Platelet-CD4+ T Cell Aggregates Are Correlated With HIV-1 Permissiveness and CD4+ T Cell Loss
Source: Front Immunol. 2021 Dec 20;12:799124. doi: 10.3389/fimmu.2021.799124 (PMC8720770; doi:10.3389/fimmu.2021.799124)
Supplement: Supplementary file 1 [file DataSheet_1.pdf]

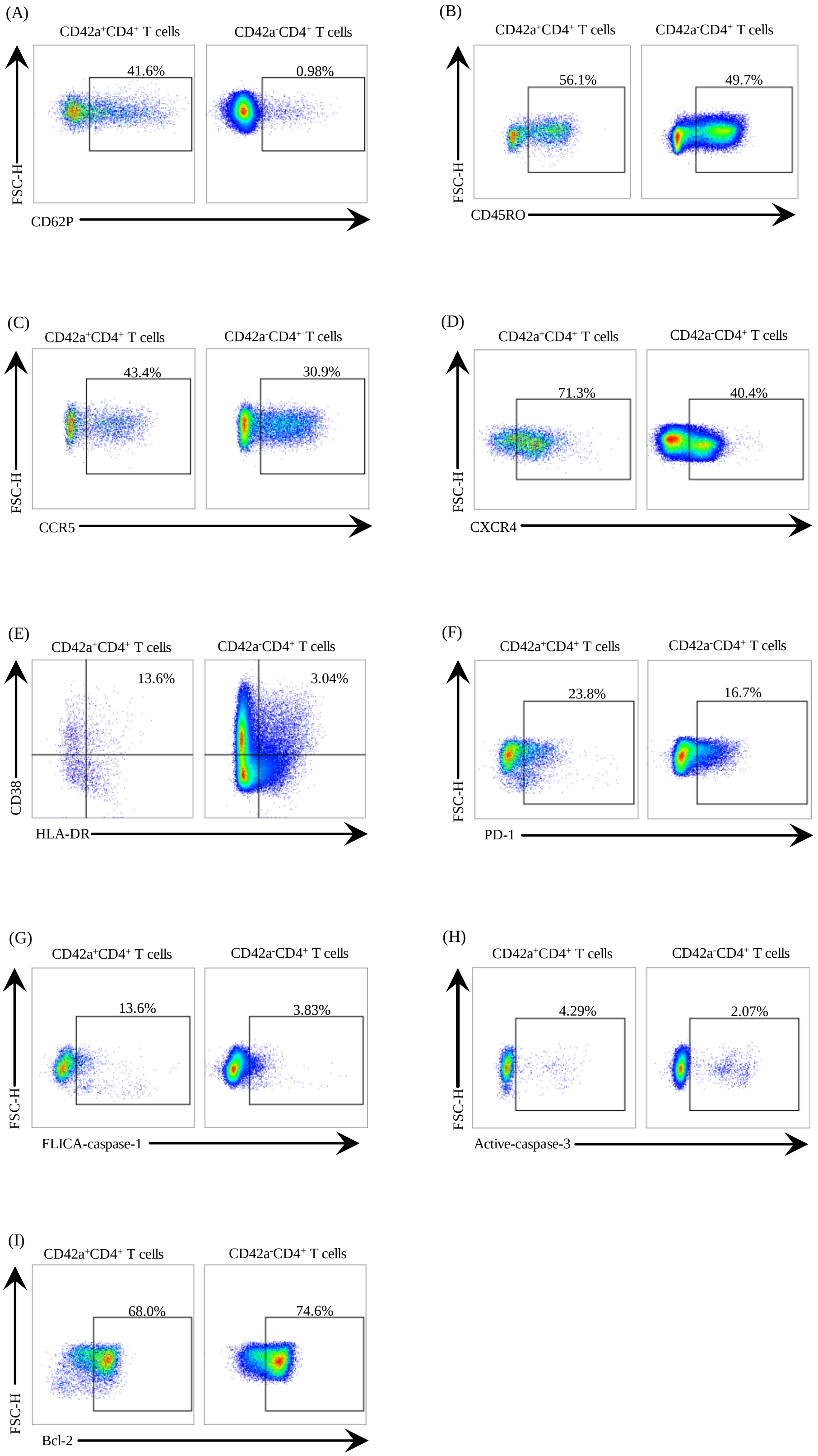

Supplementary Figure 1. Detection of phenotypic characteristics of platelet-$CD4^+$ T cell aggregates Representative gating strategy for CD62P (A), CD45RO (B), CCR5 (C), CXCR4 (D), HLA-DR/CD38 (E), PD-1 (F), FLICA-caspase-1 (G), Active-caspase-3 (H), and Bcl-2 (I) expression in platelet-$CD4^+$ T cell aggregates and their counterparts.
